# Synthetic double inversion recovery imaging in brain MRI: quantitative evaluation and feasibility of synthetic MRI and a comparison with conventional double inversion recovery and fluid-attenuated inversion recovery sequences

**DOI:** 10.1186/s12880-022-00877-4

**Published:** 2022-10-27

**Authors:** Odgerel Zorigt, Takahito Nakajima, Yuka Kumasaka, Akiko Jingu, Yoshito Tsushima

**Affiliations:** 1 Department of Diagnostic Radiology and Nuclear Medicine, Gunma University Graduate School of Medicine, 3-39-22, Showa, Maebashi, Gunma 371-9511 Japan; 2 Department of Diagnostic and Interventional Radiology, University of Tsukuba, 1-1-1, Tennoudai, Tsukuba, Ibaraki 305-8575 Japan; 3 Department of Radiology, Fujioka General Hospital, 813-1, Nakakurisu, Fujioka, Gunma 375-0015 Japan

**Keywords:** Magnetic resonance imaging, Synthetic magnetic resonance imaging, Double inversion recovery, Inversion recovery

## Abstract

**Background and purpose:**

Synthetic MR imaging (SyMRI) allows the reconstruction of various contrast images, including double inversion recovery (DIR), from a single scan. This study aimed to investigate the advantages of SyMRI by comparing synthetic DIR images with synthetic T2-weighted fluid-attenuated inversion recovery (T2W-FLAIR) and conventional DIR images.

**Materials and methods:**

We retrospectively reviewed the imaging data of 100 consecutive patients who underwent brain MRI between December 2018 and March 2019. Synthetic DIR, T2W-FLAIR, T1-weighted, and phase-sensitive inversion recovery (PSIR) images were generated from SyMRI data. For synthetic DIR, the two inversion times required to suppress white matter and cerebrospinal fluid (CSF) were manually determined by two radiologists. Quantitative analysis was performed by manually tracing the region of interest (ROI) at the sites of the lesion, white matter, and CSF. Synthetic DIR, synthetic T2W-FLAIR, and conventional DIR images were compared on the basis of using the gray matter-to-white matter, lesion-to-white matter, and lesion-to-CSF contrast-to-noise ratios.

**Results:**

The two radiologists showed no differences in setting inversion time (TI) values, and their evaluations showed excellent interobserver agreement. The mean signal intensities obtained with synthetic DIR were significantly higher than those obtained with synthetic T2W-FLAIR and conventional DIR.

**Conclusion:**

Synthetic DIR images showed a higher contrast than synthetic T2WFLAIR and conventional DIR images.

**Supplementary Information:**

The online version contains supplementary material available at 10.1186/s12880-022-00877-4.

## Introduction

MRI is widely used to evaluate intracranial pathology because of its excellent soft-tissue contrast. To clearly depict lesions in the brain, inversion recovery sequences, such as fluid-attenuated inversion recovery (FLAIR) and double inversion recovery (DIR), are often employed in clinical practice. Redpath et al. reported that the DIR sequence improved the contrast between the lesion and background areas by suppressing signals from the cerebrospinal fluid (CSF) and normal white matter (WM) [1]. DIR has been used to evaluate various lesions in multiple sclerosis, ischemic diseases, epilepsy, tumors, and degenerative diseases [2–6].

Nevertheless, the addition of DIR sequences to routine clinical practice remains challenging because of the increased examination time. Moreover, adjustment of the dual inversion time (TI_1_ and TI_2_) parameters to suit various patient conditions to selectively suppress WM and CSF may be challenging in routine clinical practice.

Synthetic magnetic resonance imaging (SyMRI) techniques can solve the problems of increased examination times and selection of appropriate TI values by allowing manual changes to the appropriate TI values after image acquisition. The image data from SyMRI examinations can be used to generate multiple contrasts from a single scan based on a quantitative approach that includes absolute physical properties, such as the longitudinal T1-relaxation time, transverse T2-relaxation time, and proton density [7]. Thus, acquisition parameters such as repetition time (TR), echo time (TE), and inversion time (TI) can be generated using mathematical reasoning rather than predetermined methods [8].

Synthetic DIR images have been generated from SyMRI image data to set null points in two different brain structures: the WM and CSF. Although appropriate TI values can be determined by drawing manual regions of interest (ROIs) in these structures, qualitative and quantitative evaluation of the generated images is essential. Therefore, this study aimed to evaluate the feasibility of SyMRI in clinical practice and investigate the advantages of SyMRI by comparing it with synthetic T2-weighted (T2W)-FLAIR and conventional DIR images.

## Materials and methods

### Patients

The Institutional Review Committee of the Fujioka General Hospital approved the study as well as the informed consent waiver (FJ-152, see Additional file 1). All methods were carried out in accordance with relevant guidelines and regulations.

**Fig. 1:**
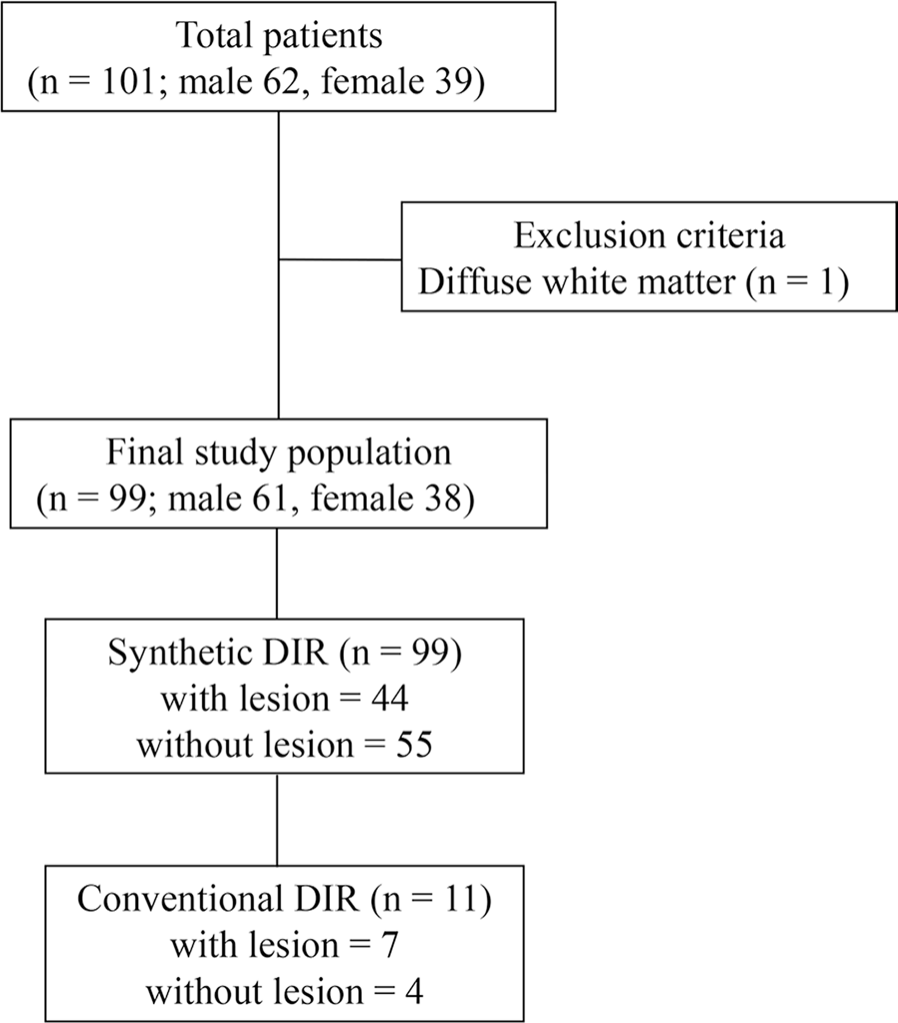
Flowchart for patient selection

One hundred consecutive patients who underwent MRI between December 2018 and March 2019 were considered for evaluation. Those ninety-nine patients’ data was used in the quantitative evaluation of synthetic MRI. One patient was excluded because of a diffuse WM lesion. Eleven patients underwent both synthetic and conventional DIR imaging. Therefore, comparison between synthetic DIR and conventional DIR were based only on cases in which both were imaged (Fig. 1).

### Image acquisition

MRI was performed using a GE Healthcare 1.5 T system with a 32-channel sensitivity encoding head coil. The routine clinical MRI protocol consisted of axial DIR and FLAIR sections. SyMRI was performed using a magnetic resonance image compilation sequence (MAGiC; GE Healthcare Japan, Tokyo, Japan), and to generate TW2-FLAIR, Phase-sensitive inversion recovery (PSIR), and T1WI, parameters such as TR, TE, and TI were determined as shown in Table 1. Imaging parameters were as follows: field of view (FOV), 220 × 220 mm; matrix, 512 × 512; receiver bandwidth, 495 kHz; slice thickness/gap, 5.0 mm/2.4 mm; and slices, 22.

**Table 1:** The parameters used for synthetic MR sequences

	TR (ms)	TE (ms)	TI (ms)
Synthetic DIR	4500	100	–
Synthetic T2W-FLAIR	15,000	100	3000
Synthetic PSIR	6000	10	500
Synthetic T1W	500	10	–

*TR* repetition time, *TE* echo time, *TI* inversion time, *DIR* double inversion recovery, *T2W-FLAIR* T2-weighted fluid-attenuated inversion recovery, *PSIR* phase-sensitive inversion recovery, *T1W* T1-weighted

### Reconstructed synthetic DIR image

**Fig. 2:**
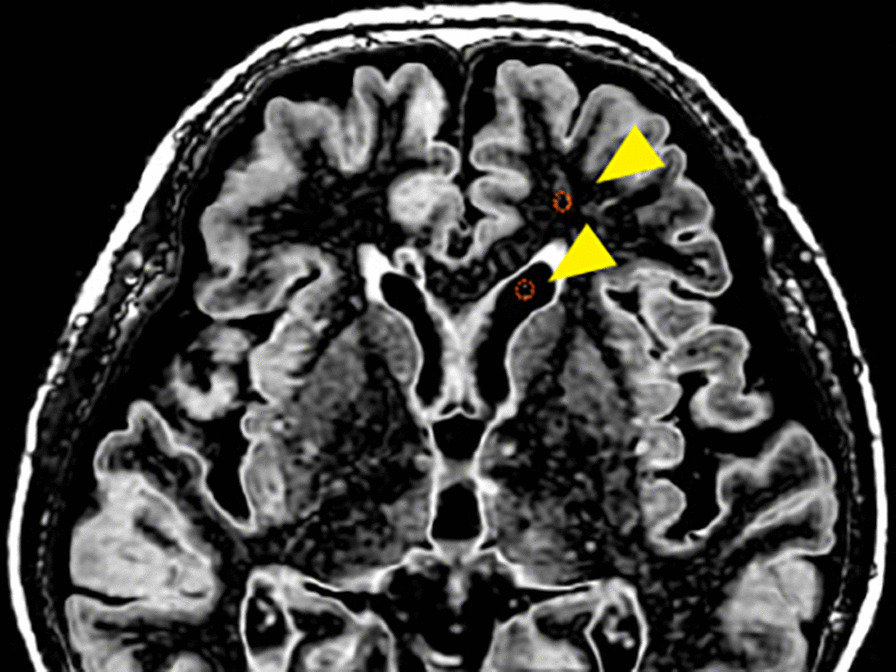
An example of tissues suppressed with ROIs placement. Circular ROIs were nulled in the CSF and WM

Circular ROIs were set to 5–10 mm by two radiologists (K. Y and J. A. with 18 and 15 years of experience, respectively). The MAGiC workstation is installed in the MR operation console. We use this software to draw ROIs and get values in each ROI. TI_1_ and TI_2_ were chosen to suppress both tissues concurrently. For TI_1_, ROIs were placed in the frontal WM, and for TI_2_, ROIs were placed in the CSF within the frontal horn of the lateral ventricle as shown in Fig. 2. ROIs were drawn on two slices at the corona radiata and corpus callosum levels.

### Quantitative image analysis

For quantitative image analysis of synMRI, ROIs with a diameter of 5 mm were placed in three constructions in the same places on different contrast images on synMRI: normal-appearing WM (genu), normal-appearing gray matter (frontal cortex), and lesions. Mean signal intensities in the ROIs were calculated using the following formula:

- SI_*GM*_/SI_*WM*_
- SI_*lesion*_/SI_*WM*_
- (SI_*GM*_/SI_*WM*_)/SI_*GM*_
- (SI_*GM*_ − SI_*WM*)_/(SI_*GM*_ + SI_*WM*_)

where SI_*GM*_, SI_*WM*_, and SI_*lesion*_ denote the signal intensities of GM, WM, and lesions, respectively.

In comparisons between synthetic DIR images and conventional DIR images, ROIs of normal-appearing WM, CSF, and lesions were set manually in the same slice and the same area. Mean signal intensities in the ROIs were calculated using the following formula:

- SI_*lesion*_/SI_*WM*_
- SI_*lesion*/_SI_*CSF*_

where SI_*CSF*_ denotes the signal intensity of CSF.

### Statistical analysis

All statistical analyses were performed using SPSS 26 for Windows (IBM Corp., Armonk, NY, USA) and GraphPad 9.0 software (GraphPad, California, USA). The differences between the TI values of the null points set by the two readers were analyzed using a nonparametric Mann–Whitney U test. Interobserver agreement between the two radiologists was determined using intraclass correlation coefficient (ICC) values. Spearman’s correlation coefficients were computed to assess the correlation between the measured TI values and age. Additionally, we determined whether statistically significant differences were present among the synthetic DIR, T2W-FLAIR, and conventional DIR images using a nonparametric Mann–Whitney U test. The mean and standard deviation were used to assess the entire population for all variables.

## Results

### Patient characteristics

A total of 99 patients (38 females and 61 males; mean age, 66 years; range, 15–95 years) were included in this study. Of the 44 patients, 18 showed periventricular hyperintensity, 9 showed chronic infractions, 6 showed acute infractions, 7 showed acute hemorrhages, 2 showed chronic hemorrhages, and the others showed malignant lymphomas and epilepsy. Seven patients with lesions were included in the comparison between synthetic and conventional DIR.

### Reliability of TI values/comparison of TI values derived from MAGiC by the two readers

**Fig. 3:**
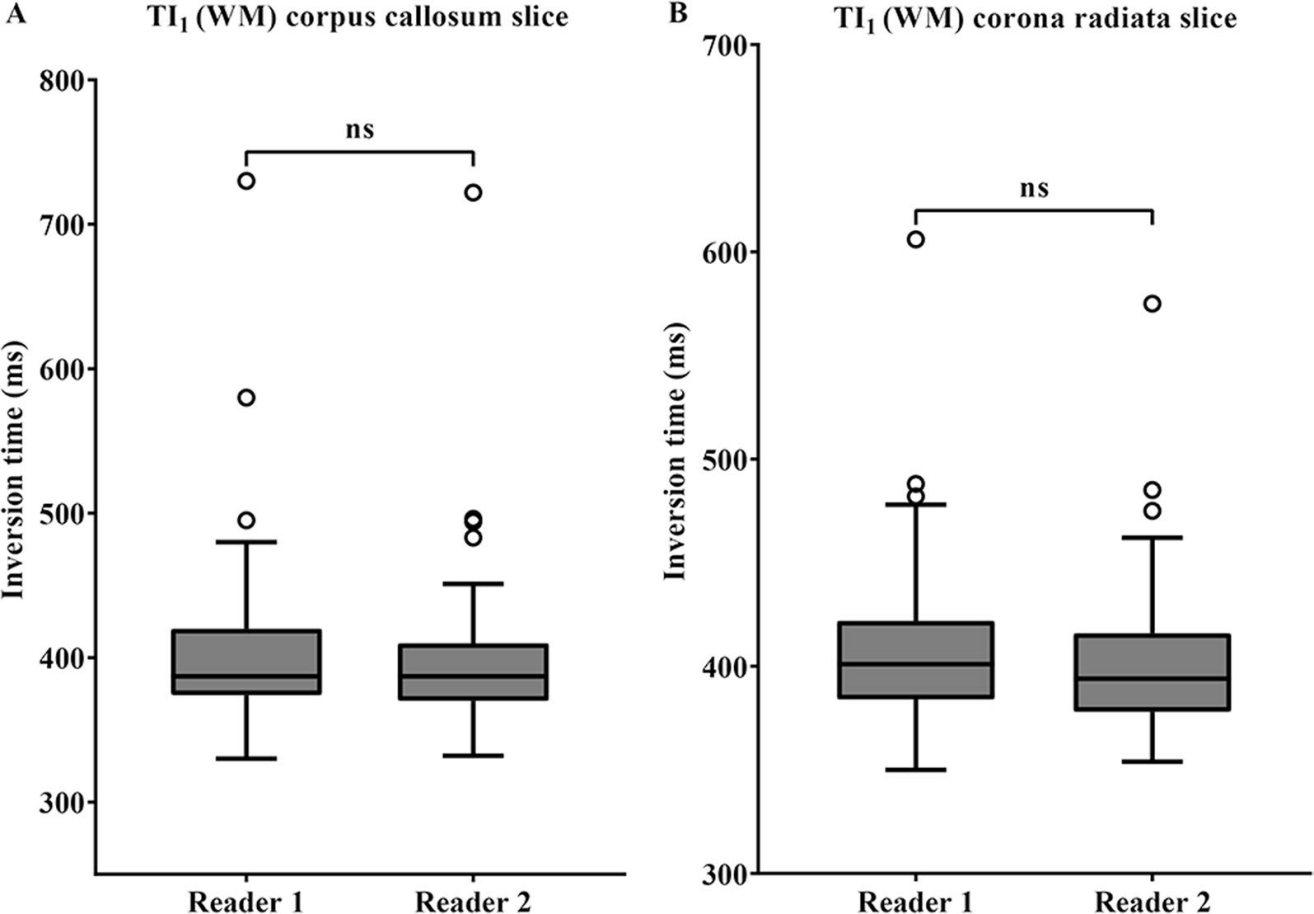
The box plot represents the comparison of TI_1_ for each slice in assessments by the two readers with WM set to null point (**A**, **B**). The TI_1_ values were placed on the two slices at the position of the corona radiata and corpus callosum. No significant difference was observed in TI_1_ between the readers. The term “ns” indicates a non-significant difference. *Note*: *TI* inversion time, *WM* white matter

**Fig. 4:**
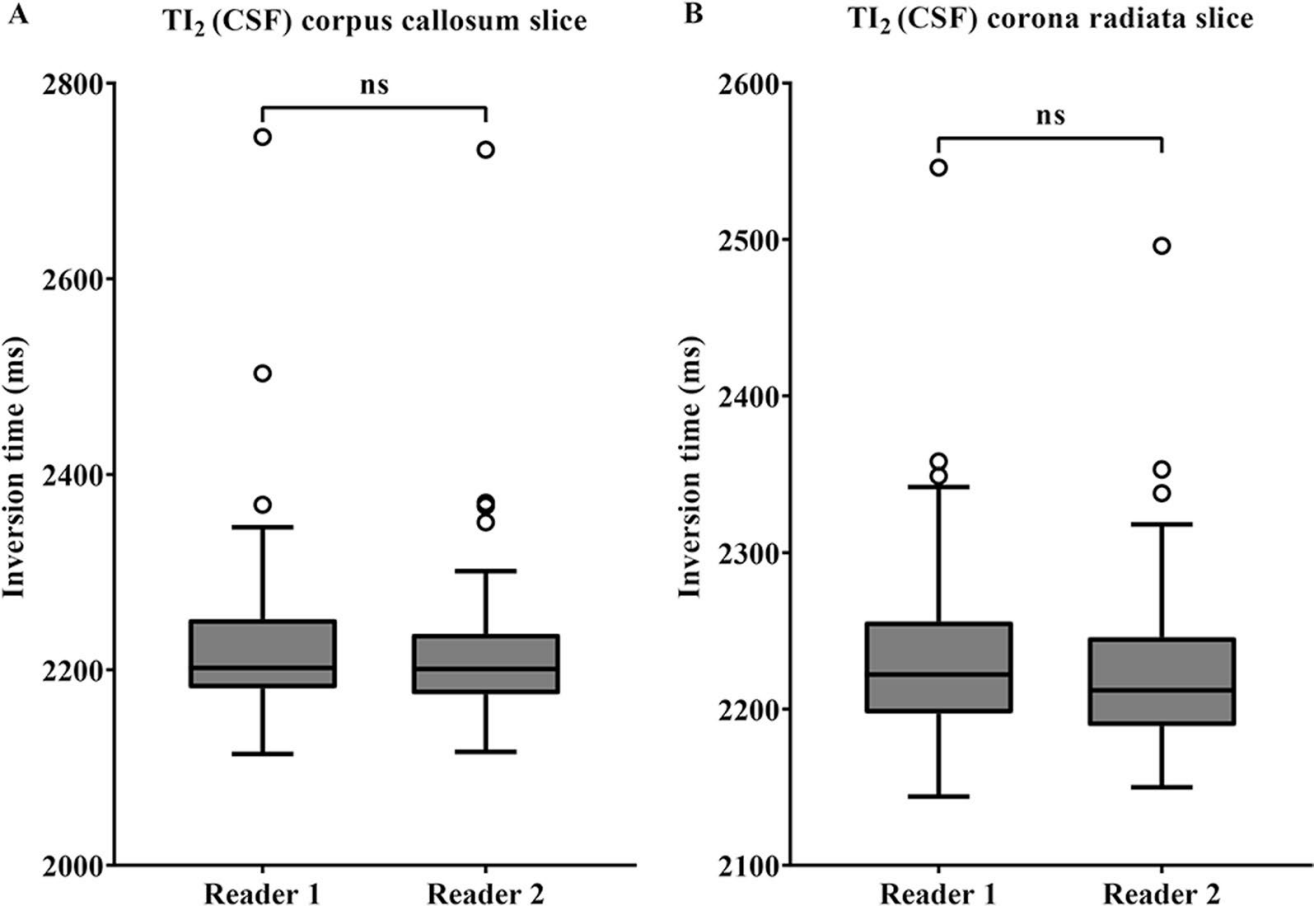
Comparison of TI_2_ for the two slices in the assessments performed by the two readers with CSF set to null point (**A**, **B**). No significant difference was observed in TI_2_ between the readers. The term “ns” indicates non-significant differences. *Note*: *TI* inversion time, *CSF* cerebrospinal fluid

A comparison of the TI values is presented in Figs. 3 and 4. No significant differences were observed in the TI_1_ values for the corpus callosum and corona radiata in comparisons between the two readers (*P* = 0.359; *P* = 0.174, Fig. 2). The average ICC of the TI_1_ value for the corpus callosum and corona radiata was 0.95 (95% CI 0.93–0.97) and 0.90 (95% CI 0.85–0.93), respectively, indicating excellent agreement between readers. For the corpus callosum and corona radiata, TI_2_ values also showed excellent agreement between the two readers, with an ICC of 0.95 (95% CI 0.93–0.97) and 0.91 (95% CI 0.86–0.93), respectively, and no significant differences (*P* = 0.338; *P* = 0.198, Fig. 3).

### Age effect

**Fig. 5:**
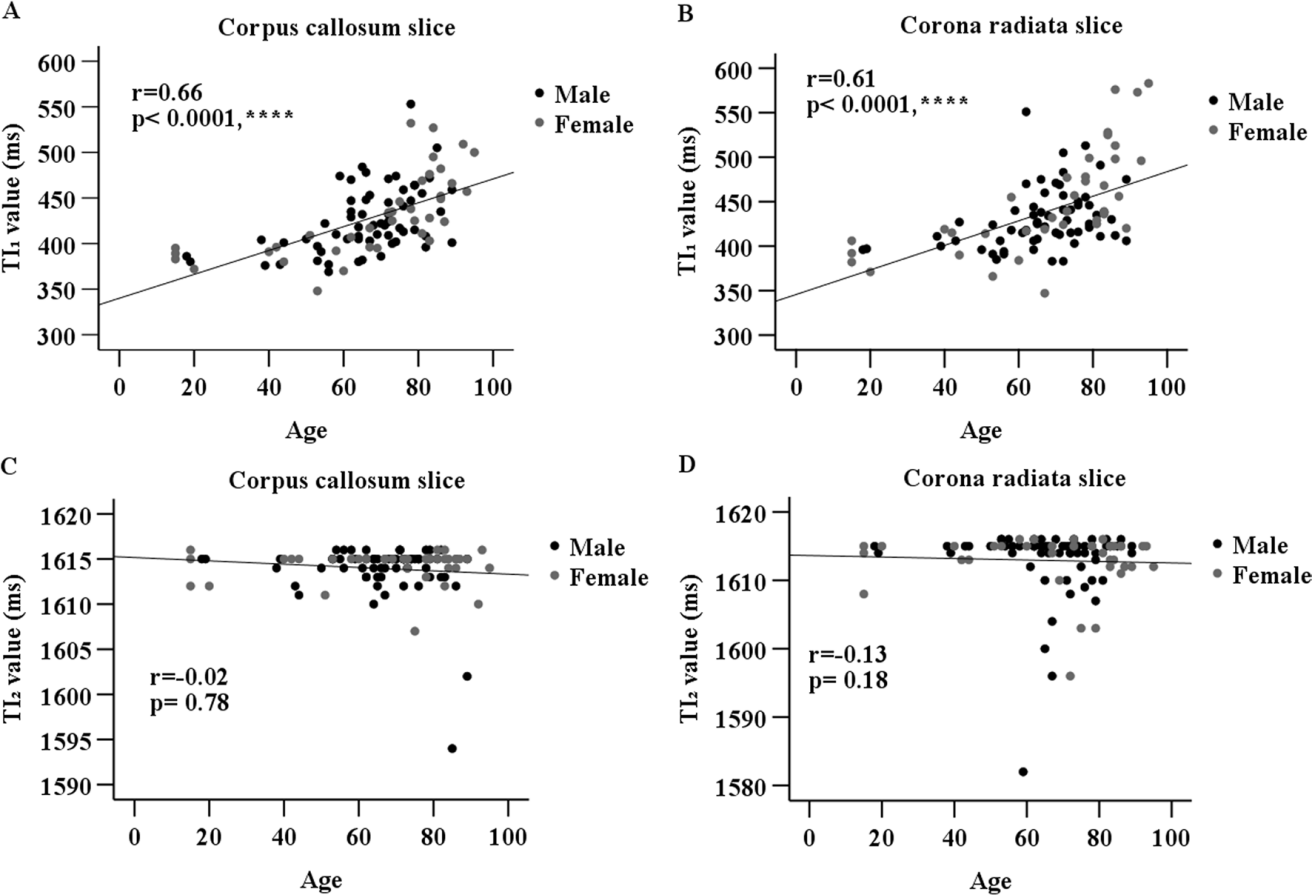
Correlation between age and TI_1_ values obtained in corona radiata image slices (**A**) and corpus callosum image slices (**B**). Correlation between age and TI_2_ values obtained in corona radiata image slices (**C**) and corpus callosum image slices (**D**). The TI_1_ values of the corona radiata image slices were moderately correlated with age (r = 0.61; *P* < 0.0001). Those of the corpus callosum image slices were also correlated (r = 0.66; *P* < 0.0001). The TI_2_ values are not significantly correlated with age in both image slice positions

Figure 5 shows the correlation between the TI values and age. TI_1_ values showed a significant correlation with age in corpus callosum slices (r = 0.66; *P* < 0.0001). The corona radiata slices also showed a significant correlation between TI_1_ values and age (r = 0.61; *P* < 0.0001). No significant correlation was observed between the TI_2_ values and age in both imaging slices.

### Quantitative image assessments

Table 2 shows a comparison between the synthetic DIR and T2W-FLAIR images. For all formulas, the values obtained with synthetic DIR images were significantly higher than those obtained with synthetic T2W-FLAIR images. The mean SI_*GM*_/SI_*WM*_ for synthetic DIR images and T2W-FLAIR images was 8.89 ± 4.93 and 1.66 ± 0.30 respectively, with significant differences (*P* < 0.001). The mean SI_*lesion*_/SI_*WM*_ for synthetic DIR images and synthetic T2W-FLAIR images was 10.78 ± 4.81 and 2.32 ± 0.43, respectively, with significant differences (*P* < 0.001). The mean (SI_*GM*_/SI_*WM*_)/SI_*GM*_ for synthetic DIR images and synthetic T2W-FLAIR images was 0.83 ± 0.13 and 0.38 ± 0.11, respectively, with significant differences (*P* < 0.001). The mean (SI_*GM*_ − SI_*WM*_)/(SI_*GM*_ + SI_*WM*_) for synthetic DIR images and synthetic T2W-FLAIR images was 0.74 ± 0.15 and 0.24 ± 0.08, respectively, with significant differences (*P* < 0.001).

**Table 2:** Comparison between synthetic DIR and synthetic T2W-FLAIR images

	Synthetic DIR	Synthetic T2W-FLAIR	p values
SI_*GM*_/SI_*WM*_	8.89 ± 4.93	1.67 ± 0.30	***
SI_*lesion*_/SI_*WM*_	10.78 ± 4.81	2.32 ± 0.43	***
(SI_*GM*_/SI_*WM*_)/SI_*GM*_	0.83 ± 0.13	0.38 ± 0.11	***
(SI_*GM*_ − SI_*WM*)_/(SI_*GM*_ + SI_*WM*)_	0.74 ± 0.15	0.24 ± 0.08	***

Data are expressed as mean and standard deviation

*SI* signal intensity, *GM* gray matter, *WM* white matter, *CSF* cerebrospinal fluid

***Statistical significances at *P* < 0.001

A comparison between synthetic and conventional DIR is shown in Table 3. Synthetic DIR images showed significantly higher values than conventional DIR images. The mean SI_*lesion*_/SI_*WM*_ for synthetic DIR images and conventional DIR images was 9.81 ± 2.81 and 5.10 ± 1.36, respectively, with significant differences (*P* < 0.01). The mean SI_*lesion*_/SI_*CSF*_ for synthetic and conventional DIR images was 23.75 ± 4.36 and 5.37 ± 1.91, respectively, with significant differences (*P* < 0.001).

**Table 3:** Comparison between synthetic DIR and conventional DIR images

	Synthetic DIR	Conventional DIR	p value
SI_*lesion*/_SI_*WM*_	9.81 ± 2.81	5.10 ± 1.36	**
SI_*lesion*_/SI*CSF*	23.75 ± 4.36	5.37 ± 1.91	***

Data are expressed as mean and standard deviation

*SI* signal intensity, *GM* gray matter, *CSF* cerebrospinal fluid

** and ***Statistical significance at *P* < 0.01, and *P* < 0.001, respectively

## Discussion

We assessed the appropriate TI values for synthetic DIR images reconstructed from conventional MRI by using MAGiC software. Several studies have reported that synthetic DIR images may be helpful in the diagnosis of brain pathologies in both adults and children [9–11]. In patients with multiple plaques, the contrast and contrast-to-noise ratios of synthetic DIR images were significantly higher than those of conventional DIR images [9]. This finding is in accordance with our results because the contrast values of synthetic DIR were higher than those of conventional DIR in our study as well.

Our quantitative image analysis demonstrated that synthetic DIR images have higher contrast than synthetic TW2-FLAIR and conventional DIR sequences. A number of publications have demonstrated the superiority of synthetic DIR in disease assessment [9–12]. Hagiwara et al. reported synthetic DIR enabled for detection of multiple sclerosis plagues. Moreover, the quality of synthetic DIR using SyMRI software is superior to conventional DIR. To our knowledge this is first image quality evaluation of synthetic DIR images reconstructed by MAGiC software. In this study, the synthetic DIR, T2W-FLAIR, T1W, and PSIR images were reconstructed from conventional MRI data obtained with a scan time of approximately 4 min and 30 s. Consequently, synthetic MRI can be evaluated by merely adjusting the parameters offline, which is desirable for time-saving examinations. Furthermore, artifacts of synthetic images are seen in the DICOM file before processing. Therefore, it has no relation to the issue after image reconstruction by the SyMRI software. [13] In the diagnosis of lesion detection, synthetic DIR images can be used to suppress certain tissues for higher lesion detection. This could be another advantage of SyMRI imaging.

In this study, the average interclass correlation coefficient of TI values between the two radiologists showed excellent agreement with synthetic DIR images. Furthermore, the two radiologists showed no statistically significant difference in setting the TI values for both slice positions. Thus, in synthetic DIR, stable and appropriate TIs can be manually changed, regardless of the assessor who determines the ROI.

Interestingly, the TI_1_ value, which is a null point for WM, significantly increased with age in this study. This feature may cause difficulty in adequately setting the TI_1_ value of the WM in conventional DIR. Leukoaraiosis may also affect age-related white matter hyperintensity (WMH) [14]. Second, the genesis of WMH may reflect a failure of the glymphatic system. In glymphatic systems, perivascular space dilation with aging has been recently shown to reflect impaired failure to eliminate interstitial fluid from the WM, leading to excessive metabolic waste and inflammation [15, 16].

Our study had several limitations. The first limitation was the small number of patients who underwent both conventional DIR and SyMRI. Second, we did not measure TI_1_ and TI_2_ in conventional DIR images. Third, there has been no investigation into the disease because this study assessed the image quality. We did not evaluate the visual appearance of the lesion for diagnosis.

In conclusion, this study shows that synthetic DIR images can be helpful not only to reduce the examination time in clinical practice but also to provide high-contrast images than conventional DIR images.

## Supplementary Information

**Additional file 1. Supplemental file S1**. Ethical approval of this study.

## Data Availability

The datasets used and/or analyzed during the current study are available from the corresponding authors on reasonable request.

## Abbreviations

FLAIR: Fluid-attenuated inversion recovery
DIR: Double inversion recovery
CSF: Cerebrospinal fluid
WM: White matter
SyMRI: Synthetic magnetic resonance imaging
TR: Repetition time
ET: Echo time
TI: Inversion time
ROI: Region of interest
T2W: T2-weighted
FOV: Field of view
MAGiC: Magnetic resonance image compilation sequence
PSIR: Phase-sensitive inversion recovery
ICC: Intraclass correlation coefficient
