# Supplementary material for: Synthetic double inversion recovery imaging in brain MRI: quantitative evaluation and feasibility of synthetic MRI and a comparison with conventional double inversion recovery and fluid-attenuated inversion recovery sequences
Source: BMC Med Imaging. 2022 Oct 27;22:183. doi: 10.1186/s12880-022-00877-4 (PMC9615305; doi:10.1186/s12880-022-00877-4)
Supplement: Supplementary file 1 — Additional file 1. Supplemental file S1. Ethical approval of this study. [file 12880_2022_877_MOESM1_ESM.pdf]

# 倫理審査結果通知書

平成３１年３月２７日

（申請者）
放射線診断科　熊坂　百香

公立藤岡総合病院
病院長　石崎政利

2019年2月21日付で申請のあった事項について、次のとおり決定したので通知します。

| | |
|---|---|
| 1　医療・医学研究等申請項名 | MRI MAGiCアプリケーションの日常臨床での使用とMaGiCを用いたWAIR画像の作成について |
| 2　審議決定事項 | 承認 |
